# Identification of Key Genes in Purine Metabolism as Prognostic Biomarker for Hepatocellular Carcinoma

**DOI:** 10.3389/fonc.2020.583053

**Published:** 2021-01-14

**Authors:** Wen-Jing Su, Pei-Zhi Lu, Yong Wu, Kumari Kalpana, Cheng-Kun Yang, Guo-Dong Lu

**Affiliations:** ^1^Department of Toxicology, School of Public Health, Guangxi Medical University, Nanning, China; ^2^Cancer Science Institute of Singapore, National University of Singapore, Singapore, Singapore; ^3^Department of Hepatobiliary Surgery, First Affiliated Hospital of Guangxi Medical University, Nanning, China; ^4^Key Laboratory of High-incidence-Tumor Prevention & Treatment (Guangxi Medical University), Ministry of Education of China, Nanning, China

**Keywords:** hepatocellular carcinoma, purine metabolism, bioinformatics, prognosis risk model, biomarker

## Abstract

**Background:**

Deregulated purine metabolism is critical for fast-growing tumor cells by providing nucleotide building blocks and cofactors. Importantly, purine antimetabolites belong to the earliest developed anticancer drugs and are still prescribed in clinics today. However, these antimetabolites can inhibit non-tumor cells and cause undesired side effects. As liver has the highest concentration of purines, it makes liver cancer a good model to study important nodes of dysregulated purine metabolism for better patient selection and precisive cancer treatment.

**Methods:**

By using a training dataset from TCGA, we investigated the differentially expressed genes (DEG) of purine metabolism pathway (hsa00230) in hepatocellular carcinoma (HCC) and determined their clinical correlations to patient survival. A prognosis model was established by Lasso‐penalized Cox regression analysis, and then validated through multiple examinations including Cox regression analysis, stratified analysis, and nomogram using another ICGC test dataset. We next treated HCC cells using chemical drugs of the key enzymes *in vitro* to determine targetable candidates in HCC.

**Results:**

The DEG analysis found 43 up-regulated and 2 down-regulated genes in the purine metabolism pathway. Among them, 10 were markedly associated with HCC patient survival. A prognostic correlation model including five genes (PPAT, DCK, ATIC, IMPDH1, RRM2) was established and then validated using the ICGC test dataset. Multivariate Cox regression analysis found that both prognostic risk model (HR = 4.703 or 3.977) and TNM stage (HR = 2.303 or 2.957) independently predicted HCC patient survival in the two datasets respectively. The up-regulations of the five genes were further validated by comparing between 10 pairs of HCC tissues and neighboring non-tumor tissues. *In vitro* cellular experiments further confirmed that inhibition of IMPDH1 significantly repressed HCC cell proliferation.

**Conclusion:**

In summary, this study suggests that purine metabolism is deregulated in HCC. The prognostic gene correlation model based on the five purine metabolic genes may be useful in predicting HCC prognosis and patient selection. Moreover, the deregulated genes are targetable by specific inhibitors.

## Introduction

It is central for all living organisms to uptake nutrients and execute metabolism. As one of the most abundant metabolic products, purines are essential for life because they provide building blocks (adenine and guanine) of DNA and RNA (1, 2). Purines are also significant components of several important biomolecules including ATP, GTP, cAMP, NADH, and coenzyme A. These biomolecules actively participate in energy production, cellular signaling pathways, redox metabolism, and fatty acid synthesis. Furthermore, purines are involved in immune responses and host-pathogen (tumor) interactions (3). There are two main purine metabolism pathways in mammalian cells, namely purine *de novo* biosynthesis pathway and complementary salvage pathway. Most cellular requirements of purines are satisfied *via* salvage pathway by recycling degraded bases (1, 2). However, rapid proliferating cells and tumor cells have higher demands of purines which are mainly fulfilled through up-regulation of purine *de novo* biosynthesis pathway. Several enzymes in this pathway further form purinosome, a dynamic multienzyme complex, to facilitate purine metabolic flux (4).

Because purines play a crucial role in tumor cell replication, purine antimetabolites (*e.g.* 6-mercaptopurine and 6-thioguanine) have been developed as the earliest anticancer drugs and are still prescribed to treat patients with acute lymphocytic leukemia, acute myeloid leukemia, and chronic myeloid leukemia (5, 6). 6-mercaptopurine and 6-thioguanine compete with purine derivatives hypoxanthine and guanine respectively to bind to hypoxanthine-guanine phosphoribosyltransferase (HGPRT), an indispensable enzyme in purine salvage pathway. These competitions and the resultant xenobiotic metabolites can repress the biosynthesis of inosine or guanine nucleotides and subsequent DNA replication. In addition, antifolates (*e.g.* methotrexate and lometrexol) are clinically applicable to treat leukemia, lymphoma, lung cancer, breast cancer, *etc*. Mechanically, the antifolates inhibit the production of 10-formyltetrahydrofolate (10-fTHF), an essential cofactor for synthesis of inosine 5’-monophosphate (IMP, the final product of purine *de novo* biosynthesis) (7). However, many of these therapeutic inhibitors affect proliferation of normal cells and result in undesirable toxicities including liver diseases, nausea, fever, and skin rashes. Therefore, there are urgent needs to identify novel regulation nodes of purine metabolism to repress oncogenesis and cancer development with minimal effects on normal cells.

**Figure 1:**
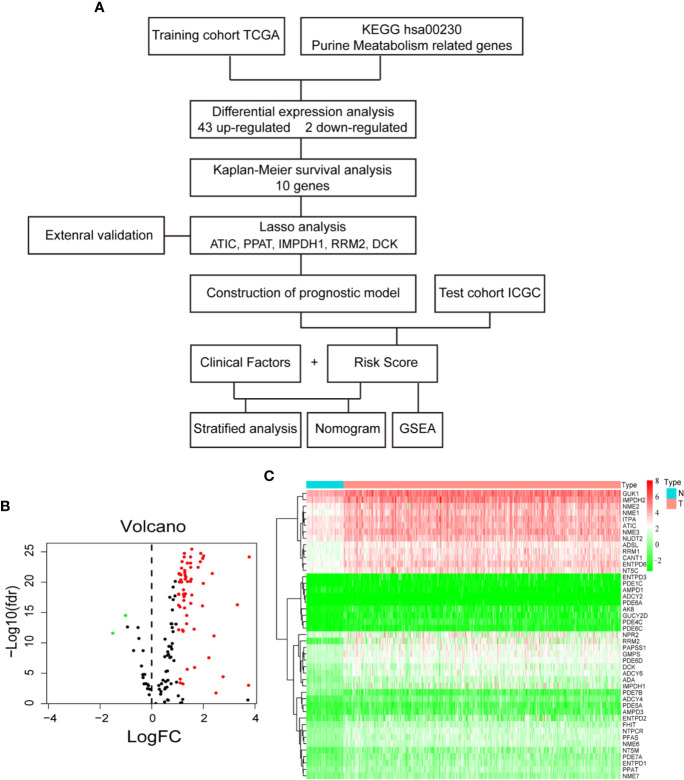
Differential gene expression analysis in TCGA database. **(A)** Flow chart of the study. **(B)** Volcano plot of differentially expressed purine metabolism genes. The red dot represents up-regulated genes, and green dot represents down-regulated genes. **(C)** Heatmap of differential gene expression.

Purines are present physiologically in the highest concentrations in liver and kidney. An important question is whether liver cancer overexpresses some targetable genes involved in purine metabolism pathway, because targeting these genes might specifically inhibit HCC but spare normal liver cells. The knowledge to be obtained will also be helpful for the treatment of other types of cancer, so that targeting these genes may induce less hepatotoxicity. Similar to other types of cancer (8–10), hepatocellular carcinoma (HCC, the predominant type of liver cancer) has deregulated purine metabolism as demonstrated by metabolomics analyses (11, 12). Notably, some serum or urine purine nucleosides were found to be useful as minimally invasive diagnostic biomarkers of HCC (11, 13). A few purine metabolic enzymes have been reported to be deregulated in HCC: *e.g.* up-regulations of a trifunctional enzyme GART (phosphoribosylglycinamide formyltransferase, phosphoribosylglycinamide synthetase, phosphoribosylaminoimidazole synthetase) (14) and a bifunctional enzyme ATIC (5-aminoimidazole-4-carboxamide ribonucleotide formyltransferase/IMP cyclohydrolase) (15) in the purine *de novo* biosynthesis pathway; and down-regulation of xanthine dehydrogenase (XDH) (16) in the purine degradation pathway. The upstream regulators of purine metabolism were recently discovered. Both mammalian target of rapamycin (mTOR) (17) and dual-specificity tyrosine phosphorylation–regulated kinase 3 (Dyrk3) (18) can activate transcription factor 4 (ATF4)-mediated transcription of methylenetetrahydrofolate dehydrogenase 2 (MTHFD2). The latter enzyme is responsible for the generation of the key cofactor 10-fTHF for IMP biosynthesis. In the present study, we explored the differentially expressed genes (DEG) involved in purine metabolism (hsa00230) and their prognostic significances in HCC by using a TCGA training datasets. A prognostic model was constructed based on five purine metabolism genes by Lasso‐penalized Cox regression analysis. The model was then validated by an ICGC test dataset. The workflow of this study was summarized in **Figure 1A**.

## Materials and Methods

### Data Collection

The training dataset of mRNA expression of 374 cases of HCC and 50 normal liver tissues were downloaded from the Cancer Genome Atlas database (TCGA, https://portal.gdc.cancer.gov/); while the corresponding clinical trait data from UCSC Xena database (http://xena.ucsc.edu/). The test dataset of gene expression and clinical trait data (the Liver Cancer-RIKEN JP) were downloaded from the International Cancer Genome Consortium (ICGC) database (https://icgc.org/). There are 130 genes in purine metabolism pathway (hsa00230) according to KEGG (https://www.kegg.jp/); and 116 genes are present in both TCGA and ICGC datasets for analyses.

### DEG Analysis and Survival Analysis

DEG analysis was performed by Wilcoxon method and then visualized by volcano plot and heatmap using the pheatmap package of R program v3.6.1 (R Foundation for Statistical Computing, Vienna, Austria). The statistical significance was set at an adjusted *P* < 0.05 and the cutoff of fold changes (FC) at log_2_FC ≥ 1 or ≤ −1. The gene expression was divided dichromatically (high or low) according to the median expression of the respective gene. The prognostic significance of DEG was determined by Kaplan-Meier method with log-rank test and the statistical significance was set at *P* < 0.01. Altogether, 343 HCC patients with more than 30 days’ follow-up in the TCGA dataset were included for the analysis.

### Construction and Validation of a Prognostic Risk Model

Lasso-penalized Cox regression analysis was utilized to construct a prognostic risk model based on the mRNA expressions of the significant prognostic genes. The model formula was constructed as: risk score = (*β*1 × mRNA1 expression) + (*β*2 × mRNA2 expression) ⋯ + (*β*n × mRNAn expression). The obtained prognostic correlation model was then validated by the ICGC test dataset which includes 229 cases of HCC patients with more than 30 days’ follow-up. The survminer package of R software was used to test the performance of the model with the optimal cutoff value determined by the surv_cutpoint function to divide the HCC patients dichromatically into high-score or low-score groups. The timeROC package was used to draw time-dependent receiver operating characteristic (ROC) curves and to evaluate the predictive value of the prognostic model.

### Multivariate Cox Regression Analyses

The independent significance of the prognostic risk model from common clinical characteristics (such as age, gender, BMI, AFP, tumor grade, TNM stage) was examined through multivariate Cox regression analyses (**Supplementary Tables 1** and **2**). Altogether, 222 cases of HCC from TCGA database and 229 HCC from ICGC database with complete clinical data were analyzed separately by the Cox analysis. *P* < 0.05 was considered statistically significant.

### Construction of a Predictive Nomogram

Nomogram is widely used for the prognosis prediction of cancer patients (19). We used the rms package of R to establish a prognostic nomogram model for assessing overall survival (OS) in HCC patients. The C index as a measure of predictive power was used to assess the performance of each model (20). Here we used the coxph function of the survival package to calculate C-index, and the lrtest function of the rms package to compare different models.

### Gene Set Enrichment Analyses (GSEA)

To explore the concomitant signaling pathways that are altered in those HCC patients with high predictive score, we performed GSEA analyses by GSEAv4.0.3 tool (http://software.broadinstitute.org/gsea/index.jsp) with KEGG gene set (c2.cp.kegg.v7.0.symbols). *P* < 0.05 and false discovery rate (FDR) <0.25 were considered as statistically significant.

### External Validations of Protein Expressions and Genetic Alterations in HCC and Examinations of Pan-cancer mRNA Expressional Changes

Next, we studied protein expressions of the candidate genes in the Human Protein Atlas database (http://www.proteinatlas.org) and genetic alterations in the cBioportal database (https://www.cbioportal.org/). The pan-cancer mRNA changes of the candidate genes were also explored in the TIMER database (https://cistrome.shinyapps.io/timer/).

### Cell Culture and Cell Viability Assay

The human HCC cell lines HepG2, SNU-398, and SNU-449 were obtained from the American Type Culture Collection (Manassas, USA), while human immortalized normal hepatic L02 cell line from China Academy of Science Shanghai Cell Bank (Shanghai, China). These cells were cultured in either RPMI 1640 medium or Dulbecco’s Modified Eagle’s medium supplemented with 10% fetal bovine serum (Gibco, USA) and 100 U/ml of penicillin and streptomycin in a 5% CO_2_ incubator at 37°C. Inhibitors against ATIC (pemetrexed), IMPDH (mycophencolate mofetil, MMF), DCK (gemcitabine), and RRM2 (osalmid) were purchased from MedChemExpress (Shanghai, China). The cell viability was determined by MTT assay (Amersco, Houston, USA) according to the manufacturer’s instructions.

### RNA Extraction and Quantitative Real-Time PCR

Total mRNA was extracted from cells using TRIZOL reagent (Invitrogen) following the manufacturer’s protocol. Equal amounts of RNA were reverse-transcribed to cDNA using the PrimeScript RT Master Mix (Takara) and analyzed by quantitative PCR (StepOnePlus; Life Technologies). β-actin and SFRS4 was used as loading control to normalize gene expression. Primer pairs used are listed below. PPAT: 5′-AGC ACC CAC AGC ATA CTC C-3′ and 5′-ACA CTG GAA TAA GAC GAC CAA TG -3′; ATIC: 5′-TTG GAG ACT AGA CGC CAG TTA-3′ and 5′-GGC ATC TGA GAT ACG CCT TTG-3′; DCK: 5′-CCA TCG AAG GGA ACA TCG CT-3′ and 5′-GGT AAA AGA CCA TCG TTC AGG T-3′; RRM2: 5′-CAC GGA GCC GAA AAC TAA AGC-3′ and 5′-TCT GCC TTC TTA TAC ATC TGC CA-3′; IMPDH1: 5′-TGA AGA AGA ACC GAG ACT ACC C-3′ and 5′-TCC AGA CGG TAT TTG TCA TCC T-3′; SFRS4: 5′- AAA AGT CGG AGC AGG AGT CA-3′ and 5′- CTC TTC CTG CCC TTC CTC TT-3′; β-actin: 5′-GGA CTT CGA GCA AGA GAT GG-3′ and 5′- AGC ACT GTG TTG GCG TAC AG-3′; SFRS4: 5’-AAA AGT CGG AGC AGG AGT CA-3, and 5’- CTC TTC CTG CCC TTC CTC TT-3’.

### Human HCC Samples

Ten pairs of dissected human primary HCCs and matched adjacent nontumor liver tissues were obtained from the Department of Hepatobiliary Surgery, First Affiliated Hospital of Guangxi Medical University, with approval from the institute review board (GXMU-20160302-10). All the 10 HCC cases are affected by HBV infection and belong to BCLC-0 or BCLC-A stages.

## Results

### DEG Analysis and Survival Analysis Using the TCGA Training Dataset

**Figure 2:**
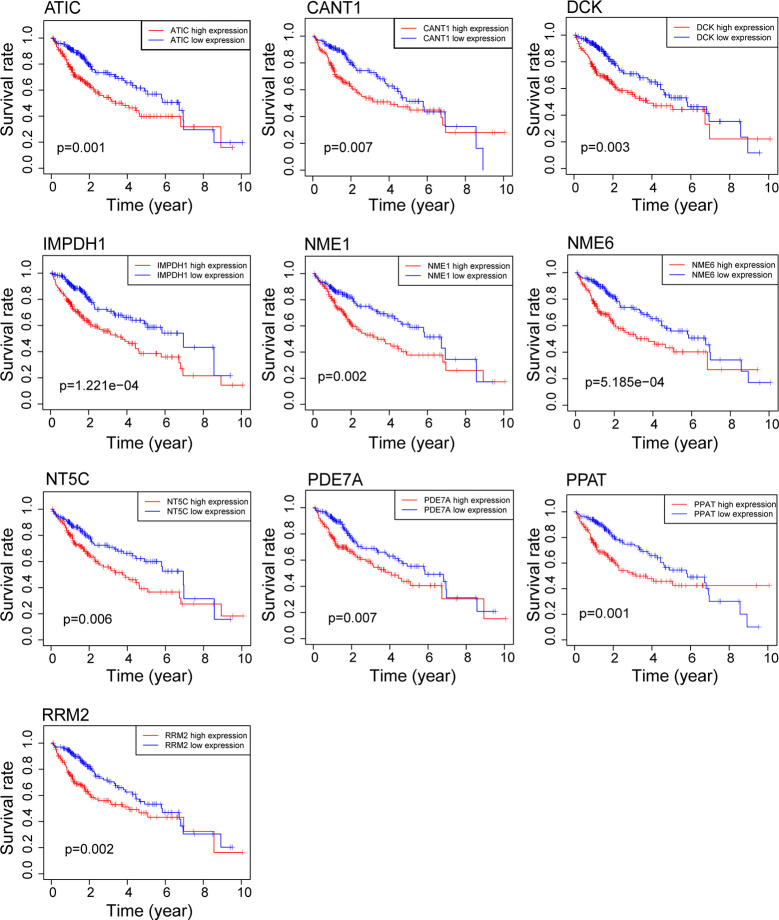
Survival analysis of genes related to purine metabolism in HCC. Kaplan-Meier survival curves of OS for 10 genes with survival significance in the TCGA database.

First, to identify the differentially expressed genes of purine metabolism pathway (KEGG hsa00230) in HCC, we compared the mRNA expressions of 374 cases of HCC with those of 50 normal liver tissues in the TCGA-LIHC dataset. Altogether, there are 43 up-regulated and 2 down-regulated genes with more than 2-fold changes (adjusted *P* < 0.05) in HCC tissues (**Figures 1B, C**). Then Kaplan-Meier survival analyses were carried out with the identified genes individually using the corresponding survival data from the UCSC Xena database. Among the 45 differentially expressed genes, 10 up-regulated genes are markedly associated with poorer patient survival (*P* < 0.01, **Figure 2**).

### Construction of a Prognostic Risk Model

Next, we applied Lasso‐penalized Cox regression analysis and identified five independent genes after removal of redundant genes (**Supplementary Figure 1**). Among the five genes, four belong to the purine *de novo* biosynthesis pathway, including ATIC [as previously reported in (15)], inosine monophosphate dehydrogenase 1 (IMPDH1), phosphoribosyl pyrophosphate amidotransferase (PPAT), and ribonucleotide reductase regulatory subunit M2 (RRM2). The fifth gene deoxycytidine kinase (DCK) predominantly converts deoxycytidine into deoxycytidine monophosphate, as one of the initial steps in the nucleotide salvage pathway.

**Figure 3:**
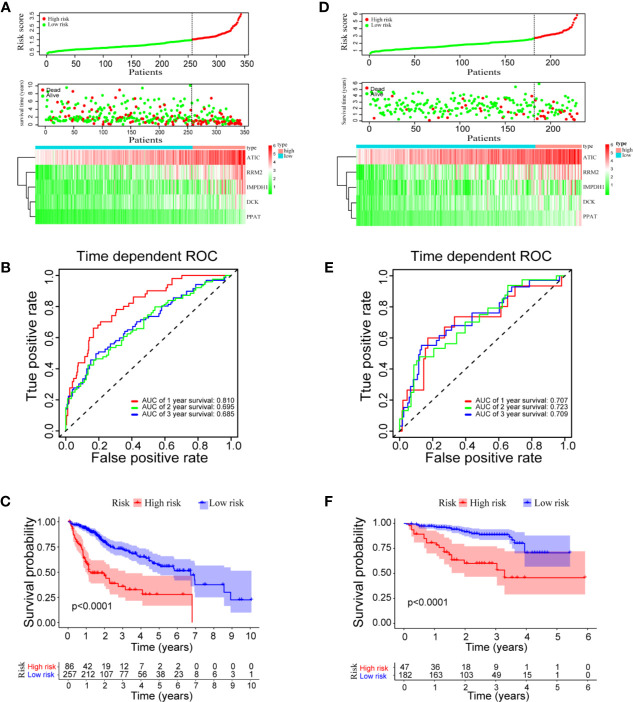
Risk score model, time-dependent ROC analysis, and survival analysis for the prognostic risk model in HCC. **(A–C)** Risk score model, time dependent ROC analysis, and survival analysis in the TCGA database, respectively. **(D–F)** Risk score model, time-dependent ROC analysis, and survival analysis in ICGC cohort, respectively.

A prognostic model was constructed based on the correlations between HCC prognosis and mRNA expressions of the five genes. The model was obtained as: risk score = 0.259 * PPAT + 0.028 * DCK + 0.026 * ATIC + 0.018 * IMPDH1 + 0.016 * RRM2. Notably, PPAT has the highest influence on the prognosis of HCC patient. As shown in **Figure 3A** upper panel, the HCC patients can be divided into high‐ and low‐risk groups with an optimal cut-off of risk score at 1.49. The patients in the high-risk group had shorter survival time, compared to those in the low-risk group (**Figure 3A** middle panel). The heatmaps of mRNA expressions of the five prognostic genes in 343 HCC patients are shown in the below panel. The ROC curves further found that area under the curve (AUC) of OS at 1-, 2-, and 3-year were 0.810, 0.695, and 0.685, respectively (**Figure 3B**). The median OS (1.2 years) of the high-risk group was significantly shorter than that of the low-risk group (6.7 years, P < 0.0001, **Figure 3C**).

### Validation of the Prognostic Model Using the ICGC Test Dataset

To validate the prognostic model in predicting HCC prognosis, the clinical HCC data from the ICGC database were used as a test dataset. Again, the HCC patients were divided into high- and low-risk groups with an optimal cutoff at 2.69 according to the prognostic risk model (**Figure 3D**). The AUC of the OS at 1-, 2-, and 3-year are 0.707, 0.723, and 0.709, respectively (**Figure 3E**). Consistent with the results using the TCGA cohort, the median OS (3.3 years) of the high-risk group in ICGC dataset is significantly shorter than that of the low-risk group (median OS not reached, *P* < 0.0001, **Figure 3F**). Overall, the results indicated a good performance of the prognostic risk model for HCC survival prediction.

To answer the question whether the mixed model of five genes is better than the respective models of single gene, we compared the time-dependent AUROC scores (**Supplementary Figure 2**). As shown in the panel A of TCGA dataset, the AUROC score of the mixed model is higher than that of individual model in year 1, year 2, and year 3. In the panel B of ICGC dataset, although the score of the mixed model is not the highest, it ranks one of the top three scores among all. More importantly, models based on single gene is not stable. In the TCGA dataset, PPAT model seems to be the best among all five selected genes. But in the ICGC dataset, RRM2 is the best. Therefore, the mixed model of five selected genes can stably predict HCC overall survival, compared to the respective models based on single gene.

### The Prognostic Risk Model Is Independent of Common HCC Clinical Characteristics

**Figure 4:**
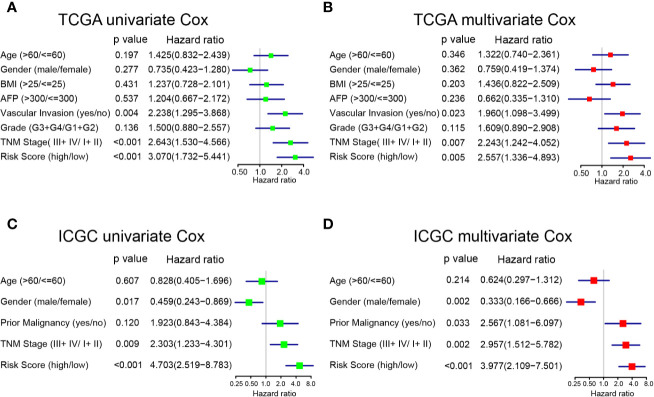
Univariate and multivariate analyses of factors associated with survival in TCGA and ICGC. **(A)** Univariate and **(B)** multivariate analysis of risk factors for overall survival in TCGA. **(C)** Univariate and **(D)** multivariate analysis of risk factors for overall survival in ICGC.

To determine whether the prognostic model was independent of common clinical characteristics in predicting HCC prognosis, we performed univariate and multivariate Cox regression analysis using the two separate TCGA and ICGC datasets. In the TCGA dataset, the prognostic model was found to have the highest hazard ratio (HR = 3.070) in univariate analyses (**Figure 4A**). More importantly, the multivariate Cox regression analysis showed that the prognostic model (HR = 2.557, 95% CI = 2.909–8.783), TNM stage (HR = 2.243), and vascular invasion (HR = 1.960) were independent risk factors of OS (**Figure 4B**). Consistent results were achieved in the ICGC dataset that the prognostic model was found to be significant in both univariate and multivariate Cox analysis (HR = 3.977, 95% CI = 2.109–7.501; **Figures 4C, D**). The prognostic risk model that was constructed based on mRNA expressions of purine metabolism genes remains to be the strongest influencer of HCC survival.

**Figure 5:**
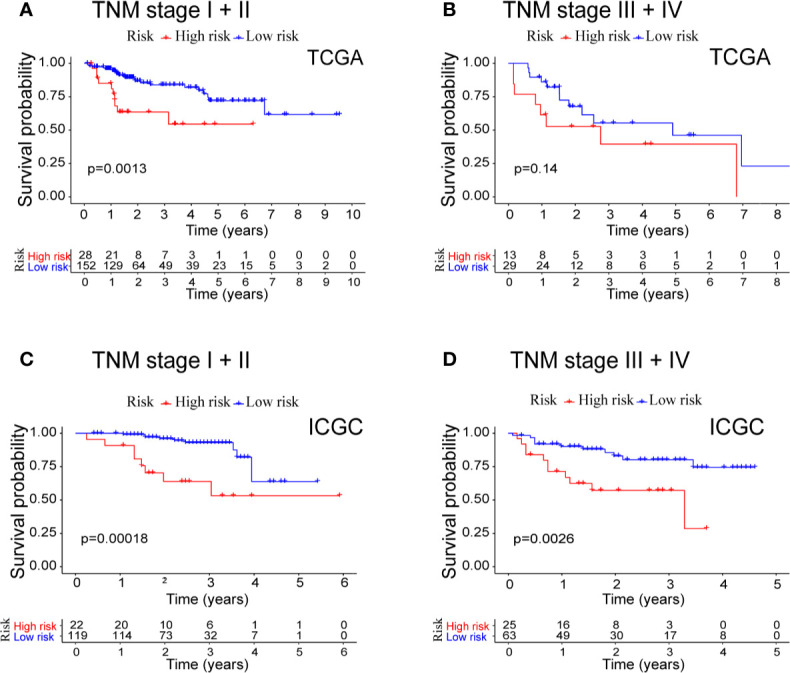
Combined survival analysis of the TNM stage and prognostic model in patients with HCC. **(A)** Kaplan−Meier survival curves of OS for high and low risk combined with TNM stage I + II in TCGA cohort. **(B)** Kaplan−Meier survival curves of OS for high and low risk combined with TNM stage III + IV in TCGA cohort. **(C)** Kaplan−Meier survival curves of OS for high and low risk combined with TNM stage I + II in ICGC cohort. **(D)** Kaplan−Meier survival curves of OS for high and low risk combined with TNM stage III + IV in ICGC cohort.

Because both the prognostic model and TNM stage are independent prognostic factors for OS in the two separate datasets, we conducted stratified analyses in order to explore whether the prognostic model was effective in predicting HCC patients with different TNM stages. In the TCGA cohort (**Figures 5A, B**), the prognostic model could differentiate OS in the HCC patients of early TNM stage (I + II, *P* = 0.0013). However, the prognostic model failed (*P* = 0.14) in those of late TNM staging (III + IV), probably because of the small sample size (N = 42). In the ICGC cohort (**Figures 5C, D**), the high-risk HCC patients differed significantly on HCC survival from the low-risk patients with either early TNM stage (*P* = 0.00018) or late TNM stage (*P* = 0.0026).

**Figure 6:**
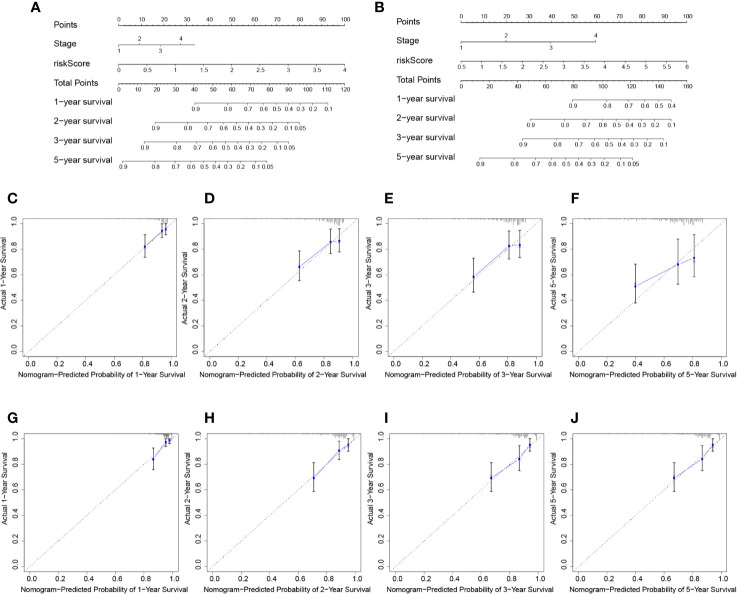
Prognostic nomogram for survival prediction. **(A)** Nomogram for overall survival in TCGA cohort. **(B)** Nomogram for overall survival in ICGC cohort. The calibration curves of overall HCC survival at 1-, 2-, and 3-years in the TCGA database **(C–F)** and ICGC database **(G–J)** are presented.

Nomogram was built by including TNM stage and the risk score in the two datasets (**Figures 6A, B**). The calibration plots for the probability of survival at 1, 2, 3, and 5 years demonstrate good agreements between the nomogram predictions and actual observations (**Figures 6C–J**). In order to further compare the effectiveness of risk score, TNM stage and the nomogram, we calculated the C-index of the risk score, TNM stage, and nomogram (**Table 1**). In the TCGA cohort, the C index of nomogram is 0.695 (95% CI = 0.611–0.780), higher than that of risk score 0.660 (95% CI = 0.580–0.740, *P* < 0.05) and TNM stage 0.603 (95% CI = 0.523–0.683, *P* < 0.05). Similar results were obtained in the ICGC cohort.

**Table 1:** C-index of risk score, TNM stage, and the nomogram.

	TCGA	*P value*	ICGC	*P value*
	C-index (95% CI)		C-index (95% CI)	
Nomogram	0.695 (0.611–0.780)	─	0.756 (0.672–0.840)	─
riskScore	0.660 (0.580–0.740)	<0.05	0.716 (0.622–0.810)	<0.05
Stage	0.603 (0.523–0.683)	<0.05	0.693 (0.613–0.773)	<0.05

### Pathway Analysis by GSEA

**Figure 7:**
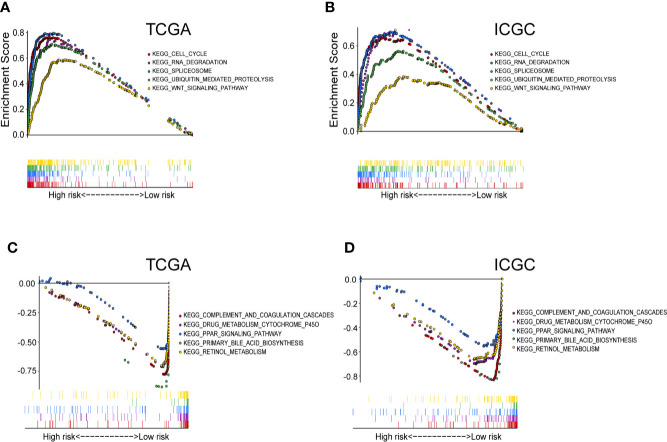
The significantly enriched KEGG pathways in TCGA and ICGC cohort by GSEA. **(A)** Five representative KEGG pathways in TCGA cohort and **(B)** ICGC cohort with high‐risk group. Five representative KEGG pathways in TCGA cohort **(C)** and ICGC cohort **(D)** with low‐risk group are listed.

To further explore the potential molecular mechanisms involved in the high-risk patients with up-regulated purine metabolism, we performed GSEA pathway analysis. We found 38 significantly enriched KEGG pathways in TCGA and ICGC cohort. Thirty-one pathways enrich in the high-risk group, including cell cycle, RNA degradation, spliceosome, WNT signaling pathway, and ubiquitin-mediated proteolysis (**Supplementary Table 3**). The top five representative pathways are shown in **Figures 7A, B**. In contrast, seven pathways enrich in the low-risk group, including complement and coagulation cascade, PPAR signaling pathway, primary bile acid biosynthesis, retinol metabolism, drug metabolism cytochrome p450 (**Supplementary Table 4**). The top five pathways are shown as well in **Figures 7C, D**.

### External Validation of the Prognostic Risk Model Using a Pancreatic Cancer Dataset

Consistent to the mRNA up-regulations, the protein levels of these five genes are also increased in HCC, compared to the normal liver tissues based on analysis using the Human Protein Atlas (**Supplementary Figure 3A**). We studied the genetic alterations among these five genes in HCC. RRM2 is the most frequently (2.5%) altered gene, with amplification as the most common alteration (**Supplementary Figure 3B**). The other four genes have less than 1% genetic alterations respectively.

Next, we determined whether the five prognostic genes were also overexpressed in other types of tumor by exploring the TIMER database. Consistent with the above results, we found that ATIC, PPAT, IMPDH1, RRM2, and DCK were all significantly overexpressed in different types of cancer (**Supplementary Figure 3C**). This pan-cancer up-regulations of the selected five genes strongly support their significance of regulatory nodes and potential cancer targets.

### IMPDH1 Inhibitor Mycophencolate Mofetil Inhibits HCC Cell Proliferation

**Figure 8:**
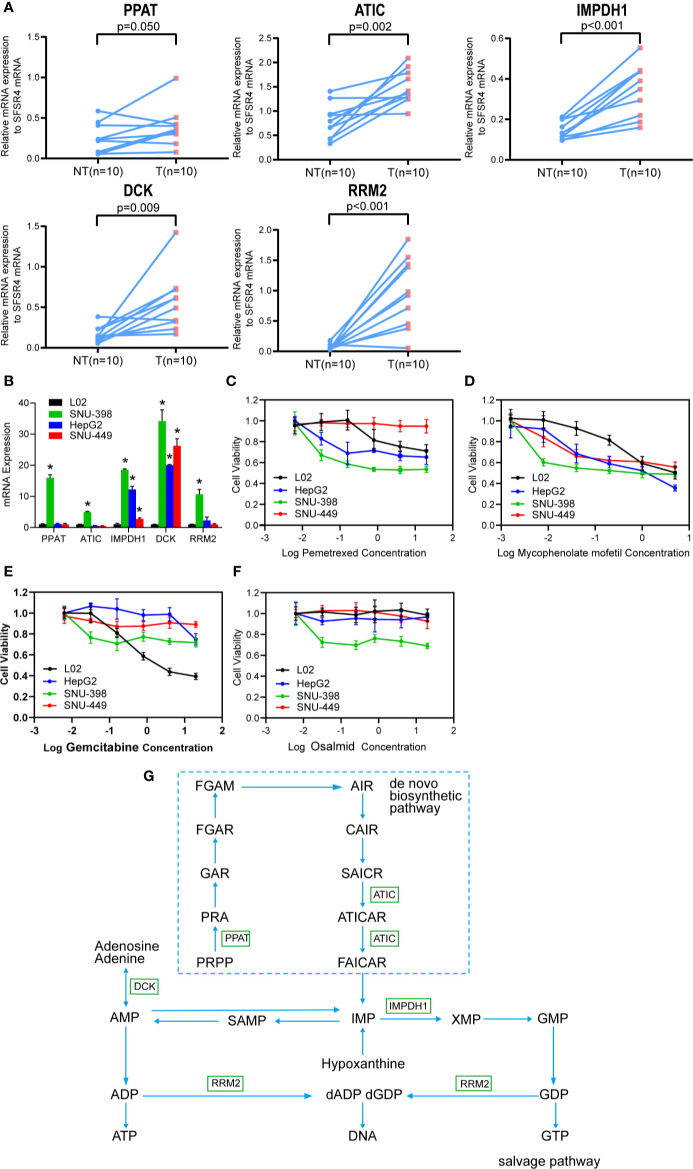
The purine metabolic genes are over-expressed in HCC and targetable specifically by IMPDH1 inhibitor. **(A)** The mRNA expression of the five selected purine metabolic genes (PPAT, ATIC, IMPDH1, DCK, and RRM2) were compared between 10 pairs of HCC tumor tissues and adjacent non-tumor liver tissues. **(B)** The mRNA expressions were also accessed in three HCC cell lines (including SNU-398, HepG2 and SNU-449) and one immortalized L02 normal hepatocyte. **(C–F)** MTT assay was used to compare the cell viability between HCC cell lines and L02 hepatocytes, after the respective treatments with increasing doses of pemetrexed **(C)**, mycophencolate mofetil **(D)**, gemcitabine **(E)**, and osalmid **(F)** for 48 h. Data were shown as the mean ± SD. **(G)** The involved purine metabolism pathway was summarized.

Lastly, the wet-lab qRT-PCR experiments compared the mRNA expressions of PPAT, ATIC, IMPDH1, DCK, and RRM2 in between 10 pairs of human primary HCC tissues and neighboring non-tumor liver tissues. The results demonstrate that these five purine metabolic genes are all up-regulated in HCC (**Figure 8A**), which are consistent with the results by the bioinformatics analyses (**Figure 1C**).

We then accessed the mRNA levels of these five genes among three HCC cells lines and one immortalized L02 hepatocyte. Compared with L02 hepatocytes which express relative low levels of these five genes, SNU-398 cells over-express all five genes while HepG2 and SNU-449 cells over-express IMPDH1 and DCK (**Figure 8B**). As expected, SNU-398 cells are sensitive to cytotoxicities of all selected inhibitors including pemetrexed against ATIC (**Figure 8C**), mycophencolate mofetil against IMPDH1 (**Figure 8D**), gemcitabine that sensitizes DCK-overexpressing cells (**Figure 8E**), and osalmid against RRM2 (**Figure 8F**). Notably, low dosages of mycophencolate mofetil are effective to inhibit all tested HCC cells lines but not the immortalized L02 hepatocytes, suggesting the potential application in HCC treatment. The rest three drugs fail to show specific inhibitions against three HCC cells but spare the immortalized L02 hepatocytes.

## Discussion

In this study, we determined the significant dysregulated genes of purine metabolism and their clinical significance in HCC. The prognostic risk model based on five purine metabolic genes (ATIC, IMPDH1, PPAT, RRM2, and DCK) reliably predicted HCC patient survival in both TCGA and ICGC datasets. The multivariate Cox regression analysis confirmed that the risk score and tumor stage were independent prognosticators in predicting HCC prognosis, apart from other common HCC clinical characteristics. These five genes are pan-cancer up-regulated. More importantly, these genes particularly IMPDH1 are targetable in cancer treatment.

The present study showed that the five genes involved in purine metabolism (**Figure 8G**) were significantly up-regulated and associated with poorer HCC prognosis. Among them, four genes (PPAT, ATIC, IMPDH1, and RRM2) belong to the purine *de novo* biosynthesis pathway which is indispensable for cancer cell proliferation (1, 2). Importantly, PPAT (with the highest coefficient in the predictive model) and ATIC (the third) are responsible for the first and final steps respectively in the pathway. PPAT catalyzes the first committed step that converts 5-phosphoribosyl-1-pyrophosphate (PRPP) into 5-phosphoribosyl-1-amine (PRA). Varambally‘s laboratory reported that PPAT and another enzyme PAICS in the purine biosynthesis pathway may drive lung adenocarcinoma cells to switch to aerobic glycolysis (the Warburg Effect) (21). This carbon-involved metabolic reprogramming directed glycolytic intermediates into purine *de novo* biosynthesis rather than TCA cycle and consequently promoted cancer cell proliferation and invasion. Notably, PPAT was over-expressed, at least partially, through genomic amplification in lung adenocarcinoma and its expression was associated with cancer aggression (21). PPAT expression by estrogen receptor *α* signaling pathway also promoted folate-mediated one-carbon metabolism and subsequent cell survival and growth in breast cancer (22). Moreover, Nakayama and coworkers recently demonstrated that PPAT played essential roles in nitrogen metabolic reprograming particularly in neuroendocrine cancer including small cell lung cancer (23). Glutamine-sourced nitrogen shifts from anaplerotic reaction into the TCA cycle to purine biosynthesis and consequently promoted cell proliferation. This novel metabolic finding may help explain earlier observations that glutamine γ-nitrogen was required for cell survival in Kaposi’s sarcoma-associated herpesvirus (KSHV) induced cancer (24). Consistently, PPAT silencing repressed cancer cell proliferation (24). Unfortunately, specific chemical inhibitor against PPAT is yet commercially unavailable.

ATIC catalyzes the final two steps in purine biosynthesis pathway with cofactor 10-fTHF to generate IMP. It has been shown that this gene was overexpressed in HCC and correlated with poorer patient prognosis (15). The authors reported that ATIC mechanically activated mTOR-S6 kinase 1 signaling. Indeed, ATIC silencing impaired HCC cell proliferation and migration (15). Recent bioinformatics studies using TCGA dataset concurred that ATIC as one of the autophagy-related genes was associated with increased cancer risks of HCC (25) and lung cancer (26). Importantly, fusion proteins of ATIC and anaplastic lymphoma kinase (ALK, a common oncogene) were frequently found in lymphoma patients (27, 28). ALK also phosphorylates ATIC at Y104 and enhances its enzymatic activity. ALK-mediated ATIC phosphorylation can rescue cancer cells from antifolate agents induced cell death (27, 29). Intriguingly, ATIC frameshift mutation and missense substitution were found in a case report who had increased sensitivity to radiation (30). Subsequent biochemical studies suggested that ATIC-involved purine biosynthesis may help DNA damage repair (30). Therefore, these results together suggested that ATIC might play an important role in carcinogenesis and cancer cell survival. Pemetrexed is an inhibitor targeting multiple purine enzymes including GART and ATIC. It repressed both ATIC-overexpressing SNU-398 cells and low-expressing HepG2 cells (**Figure 8C**). It suggests that HepG2 cells may possibly have other over-expressing enzymes that are sensitive to pemetrexed. Consistently, the ATIC-deficient SNU-449 cells are resistant to pemetrexed.

IMPDH1 catalyzes the synthesis of xanthine monophosphate (XMP) from IMP, which is a rate-limiting step to synthesize guanine nucleotides. Inhibitors of IMPDH1 *e.g.* MPA and others are currently applied clinically in the treatment of autoimmune diseases and prevention of organ transplant rejection (31, 32). IMPDH1 upregulation has been reported in many types of cancer including glioblastoma (33), colorectal cancer (34), small cell lung cancers (35), and clear cell renal cell carcinoma (36). High IMPDH1 expression was correlated with poorer patient survival in clear cell renal cell carcinoma (36). It was recently reported that IMPDH1 cooperated with metastasis-related protein Y-box binding protein 1 (YB-1) and thus promoted tumor metastasis (36). More importantly, our *in vitro* cellular study confirmed that low doses of mycophencolate mofetil were efficient to inhibit all three tested HCC cells but spare the normal L02 hepatocytes cells (**Figure 8D**). This finding warrants further preclinical studies to explore the potential application of mycophencolate mofetil in HCC treatment.

RRM2 is one of two subunits (small) of ribonucleotide reductase which catalyzes the conversion of ribonucleotides into deoxyribonucleotides. RRM2 plays an essential role in DNA synthesis, cell proliferation, and drug resistance of cancer cells. Accumulating results showed that RRM2 silencing resulted in repressed cancer cell growth and decreased drug resistance in many types of cancer including HCC (37–41). Yamada’s laboratory combined microarray analysis comparing HCC and normal liver tissues and siRNA silencing screening (41). They found RRM2 among four genes were over-expressed in HCC. More importantly, silencing of RRM2 inhibited HCC cell growth and xenograft growth. Furthermore, a recent study showed that sorafenib (the first-line therapeutic drug of HCC) targeted RRM2 by decreasing its expression (42). RRM2 overexpression partially rescued HCC cells from sorafenib-induced reduction of colony formation. Interestingly, both RRM2 and DCK are reported to be associated with the drug sensitivity of gemcitabine (a common chemotherapeutic drug for pancreatic cancer and many other types of cancer). DCK is a key rate-limiting enzyme in the nucleoside salvage pathway. It promotes the conversion of chemotherapeutic deoxyribonucleoside prodrugs to nucleoside triphosphate derivatives which execute stronger cytotoxic effects. DCK activity is associated with increased chemosensitivity to gemcitabine, contrary to the pro-chemoresistance effect of RRM2. Thus, it has been proposed that the expressional ratio of DCK to RRM2 was a predictive marker for the efficacy of gemcitabine treatment (43, 44). Recent bioinformatic studies also showed that DCK was over-expressed in HCC and correlated with increased infiltration of immune cells but with poorer patient survival (45, 46). However, in the present study, both DCK and RRM2 were found to be over-expressed in HCC (**Figure 8A**). The cytotoxicity studies showed that only SNU-398 cells that over-express RRM2 were sensitive to the inhibitor osalmid (**Figure 8F**). Consistently, the two low-expressing HepG2 and SNU-449 cell lines, together with the immortalized L02 cells are resistant. However, gemcitabine failed to differentiate DCK-overexpressing HCC cell lines from the immortalized L02 hepatocytes.

Our research also has certain limitations. The TCGA database lacks clinical outcome variables about tumor progression and post-surgery clinical data, such as vascular invasion status, tumor size, HCC recurrence, and post-surgery treatments. Due to the incompleteness of clinical data, some cases in the TCGA dataset were excluded for nomogram analysis. It may possibly affect the statistical power. Thus, our study cannot rule out that the patient survival may be affected by postoperative treatments or by other critical clinical factors. Further, we only used online database analysis. The usefulness of the prognostic model in the real world is yet unclear. Future cohort studies are necessary to validate the predictive value of the model in HCC. Lastly, whether these five genes are targetable in HCC or other types of cancer is unknown. Although we showed that mycophencolate mofetil (IMPDH1 inhibitor) could inhibit HCC cell proliferation, whether a cocktail containing inhibitors against some or all five genes is useful and safe to treat patients with HCC (a highly heterogenous cancer) is yet to be tested. Therefore, future preclinical experiments and clinical studies are needed to evaluate the predictive value of the prognostic model and to test whether this prediction could be used to guide HCC treatment.

## Conclusion

Our study suggests that purine metabolism is deregulated in HCC. A prognostic gene correlation model consisting of five purine metabolic genes (PPAT, DCK, ATIC, IMPDH1, and RRM2) may be useful in predicting the prognosis of HCC. Mycophencolate mofetil (an IMPDH1 inhibitor) could inhibit HCC cell proliferation *in vitro*, suggesting that the genes in the predictive model are a potential therapeutic target in HCC. However, these results need to be verified by future preclinical and clinical studies.

## Data Availability Statement

The datasets presented in this study can be found in online repositories. The names of the repository/repositories and accession number(s) can be found in the article/**Supplementary Material**.

## Ethics Statement

The studies involving human participants were reviewed and approved by Guangxi Medical University Institute Review Board (GXMU-20160302-10). The patients/participants provided their written informed consent to participate in this study.

## Author Contributions

WJS, PZL, and YW equally performed the majority of works. WJS and PZL performed bioinformatic analyses. YW and PZL conducted wet-lab experiments. CKY provided bioinformatics technical support. YW and KK prepared the figures. KK proofread the manuscript. GDL designed the research and summarized the data. WJS and GDL wrote the paper. All authors contributed to the article and approved the submitted version.

## Funding

This work was supported by the National Natural Science Foundation of China (81672370 and 81972291), and the Guangxi Natural Science Foundation Key Grant (2018GXNSFDA050006), and the Guangxi Medical University Training Program for Distinguished Young Scholars (2017).

## Conflict of Interest

The authors declare that the research was conducted in the absence of any commercial or financial relationships that could be construed as a potential conflict of interest.
