# Supplementary material for: Identification of Key Genes in Purine Metabolism as Prognostic Biomarker for Hepatocellular Carcinoma
Source: Front Oncol. 2021 Jan 14;10:583053. doi: 10.3389/fonc.2020.583053 (PMC7841304; doi:10.3389/fonc.2020.583053)
Supplement: Supplementary file 1 [file DataSheet_1.docx]

**Supplementary Table 1.** Clinical characteristics of patients in TCGA (n=222).

| Variables | n | % | HR(95%CI) | P value |
| --- | --- | --- | --- | --- |
| Age |  |  |  |  |
| >60 | 104 | 0.47 | 1.425(0.832−2.439) | 0.197 |
| ≤60 | 118 | 0.53 |  |  |
| Gender |  |  |  |  |
| male | 153 | 0.69 | 0.735(0.423−1.280) | 0.277 |
| female | 69 | 0.31 |  |  |
| BMI |  |  |  |  |
| >25 | 106 | 0.48 | 1.237(0.728−2.101) | 0.431 |
| ≤25 | 116 | 0.52 |  |  |
| AFP |  |  |  |  |
| >300 | 54 | 0.24 | 1.204(0.667−2.172) | 0.537 |
| ≤300 | 168 | 0.76 |  |  |
| Vascular Invasion |  |  |  |  |
| yes | 76 | 0.34 | 2.238(1.295−3.868) | 0.004 |
| no | 146 | 0.66 |  |  |
| Grade |  |  |  |  |
| G3+G4 | 100 | 0.45 | 1.500(0.880−2.557) | 0.136 |
| G1+G2 | 122 | 0.55 |  |  |
| TNM stage |  |  |  |  |
| Stage III+ IV | 42 | 0.19 | 2.643(1.530−4.566) | <0.001 |
| Stage I+ II | 180 | 0.81 |  |  |
| Risk |  |  |  |  |
| high | 41 | 0.18 | 3.070(1.732−5.441) | <0.001 |
| low | 181 | 0.82 |  |  |

**Supplementary Table 2.** Clinical characteristics of patients in ICGC (n=229).

| Variables | n | % | HR(95%CI) | P value |
| --- | --- | --- | --- | --- |
| Age |  |  |  |  |
| >60 | 180 | 0.79 | 0.828(0.405−1.696) | 0.607 |
| ≤60 | 49 | 0.21 |  |  |
| Gender |  |  |  |  |
| male | 168 | 0.73 | 0.459(0.243−0.869) | 0.017 |
| female | 61 | 0.27 |  |  |
| Prior Malignancy |  |  |  |  |
| yes | 30 | 0.13 | 1.923(0.843−4.384) | 0.120 |
| no | 199 | 0.87 |  |  |
| TNM stage |  |  |  |  |
| Stage III+ IV | 88 | 0.38 | 2.303(1.233−4.301) | 0.009 |
| Stage I+ II | 141 | 0.62 |  |  |
| Risk |  |  |  |  |
| high | 47 | 0.21 | 4.703(2.519−8.783) | <0.001 |
| low | 182 | 0.79 |  |  |

**Supplementary Table 3.** Gene set enrichment analysis for high‐risk group with KEGG pathway gene sets gene symbols using the TCGA and ICGC datasets.

|  | **TCGA** | | | | | **ICGC** | | | | |
| --- | --- | --- | --- | --- | --- | --- | --- | --- | --- | --- |
| **NAME** | **SIZE** | **ES** | **NES** | **NOM p-val** | **FDR q-val** | **SIZE** | **ES** | **NES** | **NOM p-val** | **FDR q-val** |
| KEGG_PROGESTERONE_MEDIATED_OOCYTE_MATURATION | 85 | 0.68 | 2.06 | 0.00 | 0.00 | 85 | 0.50 | 1.81 | 0.00 | 0.06 |
| KEGG_CELL_CYCLE | 124 | 0.76 | 2.01 | 0.00 | 0.00 | 124 | 0.65 | 2.13 | 0.00 | 0.01 |
| KEGG_RNA_DEGRADATION | 59 | 0.74 | 2.01 | 0.00 | 0.00 | 52 | 0.71 | 2.22 | 0.00 | 0.00 |
| KEGG_SPLICEOSOME | 127 | 0.79 | 2.02 | 0.00 | 0.00 | 122 | 0.70 | 2.12 | 0.00 | 0.00 |
| KEGG_PYRIMIDINE_METABOLISM | 98 | 0.66 | 2.07 | 0.00 | 0.00 | 96 | 0.57 | 2.01 | 0.00 | 0.01 |
| KEGG_UBIQUITIN_MEDIATED_PROTEOLYSIS | 134 | 0.71 | 2.01 | 0.00 | 0.00 | 130 | 0.56 | 2.03 | 0.00 | 0.01 |
| KEGG_VASOPRESSIN_REGULATED_WATER_REABSORPTION | 44 | 0.72 | 2.05 | 0.00 | 0.00 | 43 | 0.52 | 1.68 | 0.01 | 0.13 |
| KEGG_PURINE_METABOLISM | 158 | 0.61 | 2.15 | 0.00 | 0.00 | 149 | 0.49 | 2.00 | 0.00 | 0.01 |
| KEGG_BLADDER_CANCER | 42 | 0.67 | 1.98 | 0.00 | 0.00 | 40 | 0.49 | 1.60 | 0.01 | 0.16 |
| KEGG_NUCLEOTIDE_EXCISION_REPAIR | 44 | 0.76 | 1.97 | 0.00 | 0.00 | 44 | 0.65 | 1.98 | 0.00 | 0.01 |
| KEGG_BASE_EXCISION_REPAIR | 35 | 0.75 | 1.94 | 0.00 | 0.00 | 33 | 0.68 | 2.05 | 0.00 | 0.01 |
| KEGG_OOCYTE_MEIOSIS | 112 | 0.70 | 2.15 | 0.00 | 0.00 | 110 | 0.55 | 2.06 | 0.00 | 0.01 |
| KEGG_AMINOACYL_TRNA_BIOSYNTHESIS | 41 | 0.73 | 1.89 | 0.00 | 0.00 | 41 | 0.67 | 1.89 | 0.01 | 0.03 |
| KEGG_THYROID_CANCER | 29 | 0.68 | 1.90 | 0.00 | 0.01 | 29 | 0.48 | 1.62 | 0.02 | 0.15 |
| KEGG_PATHOGENIC_ESCHERICHIA_COLI_INFECTION | 56 | 0.67 | 1.89 | 0.00 | 0.01 | 53 | 0.55 | 1.77 | 0.01 | 0.08 |
| KEGG_WNT_SIGNALING_PATHWAY | 150 | 0.59 | 1.90 | 0.00 | 0.01 | 149 | 0.38 | 1.52 | 0.03 | 0.22 |
| KEGG_ADHERENS_JUNCTION | 73 | 0.66 | 1.91 | 0.00 | 0.01 | 68 | 0.47 | 1.63 | 0.01 | 0.17 |
| KEGG_COLORECTAL_CANCER | 62 | 0.66 | 1.88 | 0.00 | 0.01 | 62 | 0.49 | 1.73 | 0.01 | 0.10 |
| KEGG_CHRONIC_MYELOID_LEUKEMIA | 73 | 0.67 | 1.90 | 0.00 | 0.01 | 73 | 0.47 | 1.63 | 0.02 | 0.16 |
| KEGG_HOMOLOGOUS_RECOMBINATION | 28 | 0.79 | 1.87 | 0.00 | 0.01 | 26 | 0.71 | 1.92 | 0.00 | 0.02 |
| KEGG_SMALL_CELL_LUNG_CANCER | 84 | 0.62 | 1.85 | 0.00 | 0.01 | 84 | 0.43 | 1.55 | 0.03 | 0.20 |
| KEGG_ENDOMETRIAL_CANCER | 52 | 0.64 | 1.86 | 0.00 | 0.01 | 52 | 0.44 | 1.57 | 0.04 | 0.18 |
| KEGG_NON_SMALL_CELL_LUNG_CANCER | 54 | 0.64 | 1.86 | 0.00 | 0.01 | 54 | 0.48 | 1.69 | 0.00 | 0.13 |
| KEGG_DNA_REPLICATION | 36 | 0.83 | 1.85 | 0.00 | 0.01 | 36 | 0.78 | 1.95 | 0.00 | 0.02 |
| KEGG_N_GLYCAN_BIOSYNTHESIS | 46 | 0.64 | 1.84 | 0.00 | 0.01 | 46 | 0.55 | 1.79 | 0.02 | 0.08 |
| KEGG_BASAL_TRANSCRIPTION_FACTORS | 35 | 0.69 | 1.85 | 0.00 | 0.01 | 35 | 0.56 | 1.78 | 0.00 | 0.08 |
| KEGG_RENAL_CELL_CARCINOMA | 70 | 0.62 | 1.84 | 0.00 | 0.01 | 66 | 0.48 | 1.63 | 0.03 | 0.16 |
| KEGG_RNA_POLYMERASE | 29 | 0.69 | 1.82 | 0.00 | 0.01 | 29 | 0.62 | 1.75 | 0.02 | 0.09 |
| KEGG_PANCREATIC_CANCER | 70 | 0.66 | 1.82 | 0.00 | 0.01 | 69 | 0.50 | 1.67 | 0.01 | 0.14 |
| KEGG_MISMATCH_REPAIR | 23 | 0.79 | 1.79 | 0.00 | 0.01 | 23 | 0.74 | 1.92 | 0.00 | 0.02 |
| KEGG_PROSTATE_CANCER | 89 | 0.56 | 1.75 | 0.00 | 0.02 | 89 | 0.40 | 1.51 | 0.03 | 0.22 |

**Supplementary Table 4.** Gene set enrichment analysis for low‐risk group with KEGG pathway gene sets gene symbols using the TCGA and ICGC datasets。

|  | **TCGA** | | | | | **ICGC** | | | | |
| --- | --- | --- | --- | --- | --- | --- | --- | --- | --- | --- |
| **NAME** | **SIZE** | **ES** | **NES** | **NOM p-val** | **FDR q-val** | **SIZE** | **ES** | **NES** | **NOM p-val** | **FDR q-val** |
| KEGG_COMPLEMENT_AND_COAGULATION_CASCADES | 69 | -0.78 | -2.14 | 0.00 | 0.00 | 69 | -0.84 | -2.29 | 0.00 | 0.00 |
| KEGG_GLYCINE_SERINE_AND_THREONINE_METABOLISM | 31 | -0.78 | -2.00 | 0.00 | 0.00 | 31 | -0.67 | -1.69 | 0.03 | 0.14 |
| KEGG_PRIMARY_BILE_ACID_BIOSYNTHESIS | 16 | -0.92 | -2.01 | 0.00 | 0.00 | 16 | -0.87 | -1.94 | 0.00 | 0.04 |
| KEGG_RETINOL_METABOLISM | 64 | -0.71 | -2.02 | 0.00 | 0.00 | 63 | -0.67 | -1.81 | 0.02 | 0.06 |
| KEGG_DRUG_METABOLISM_CYTOCHROME_P450 | 71 | -0.72 | -2.02 | 0.00 | 0.00 | 72 | -0.71 | -1.91 | 0.01 | 0.03 |
| KEGG_PPAR_SIGNALING_PATHWAY | 69 | -0.57 | -1.77 | 0.01 | 0.02 | 69 | -0.56 | -1.81 | 0.01 | 0.07 |
| KEGG_LINOLEIC_ACID_METABOLISM | 29 | -0.55 | -1.65 | 0.01 | 0.04 | 29 | -0.66 | -1.92 | 0.00 | 0.04 |
