# Supplementary figures and images for: Identification of Key Genes in Purine Metabolism as Prognostic Biomarker for Hepatocellular Carcinoma
Source: Front Oncol. 2021 Jan 14;10:583053. doi: 10.3389/fonc.2020.583053 (PMC7841304; doi:10.3389/fonc.2020.583053)

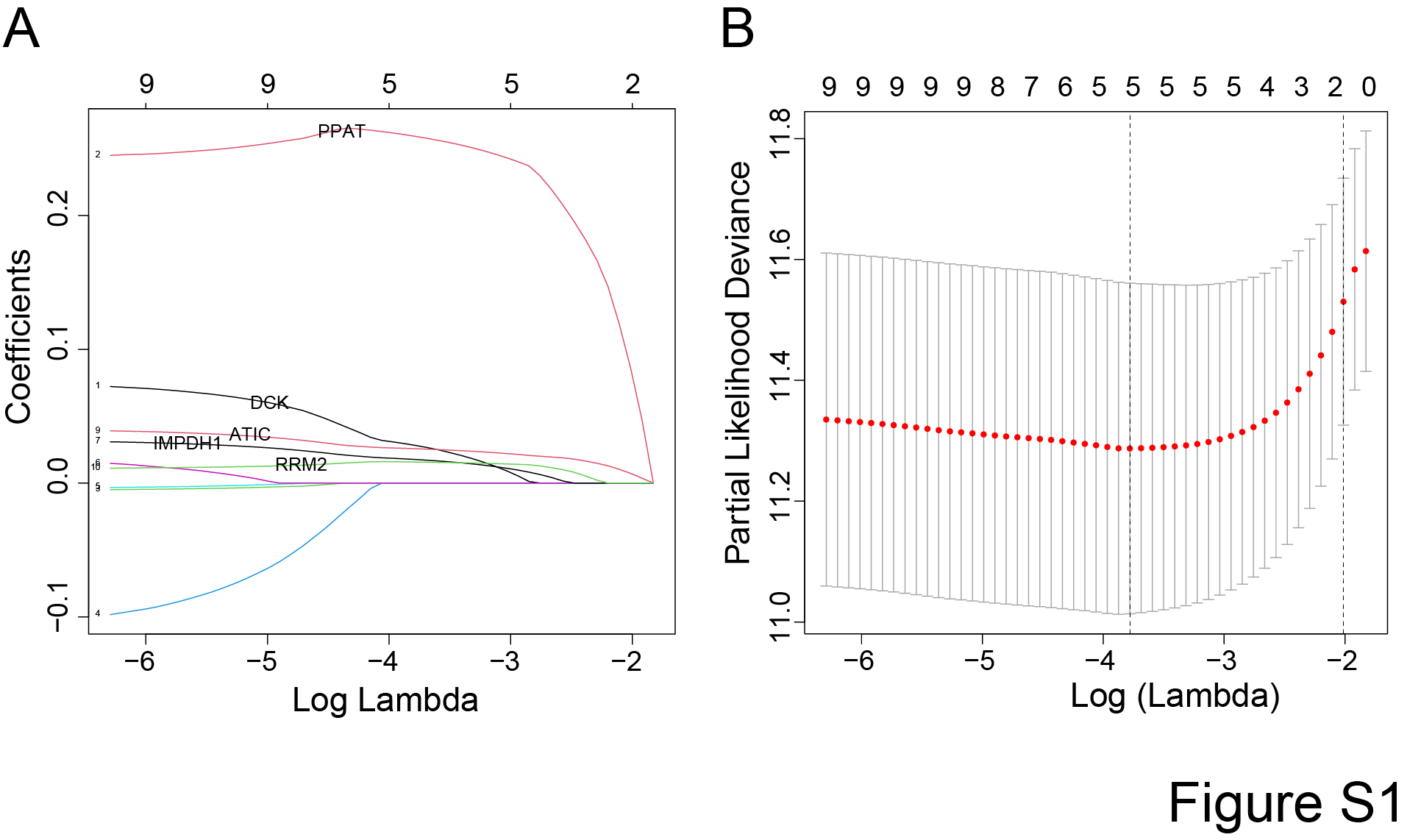

Supplement: Supplementary file 2 [file Image_1.tif]

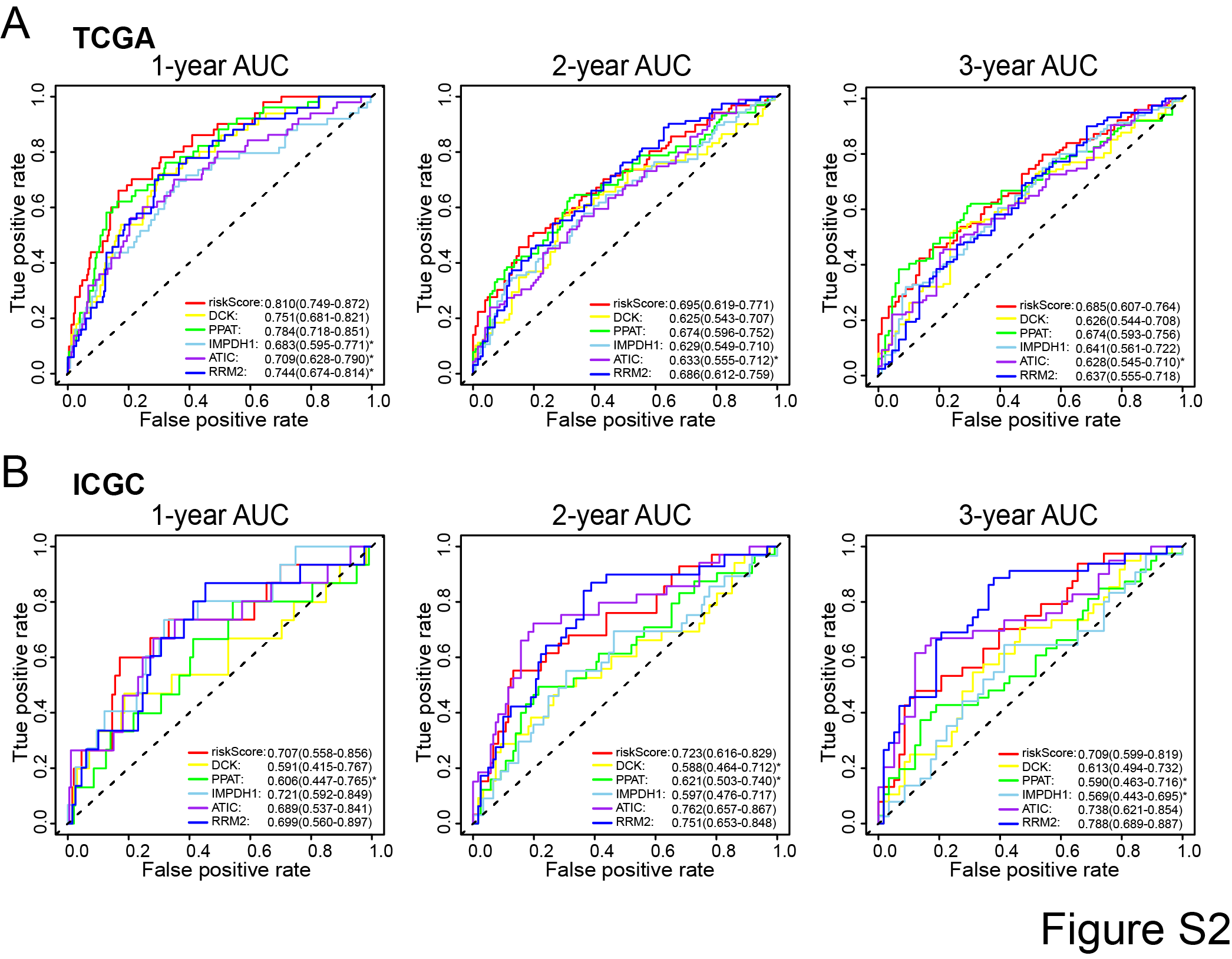

Supplement: Supplementary file 3 [file Image_2.tif]

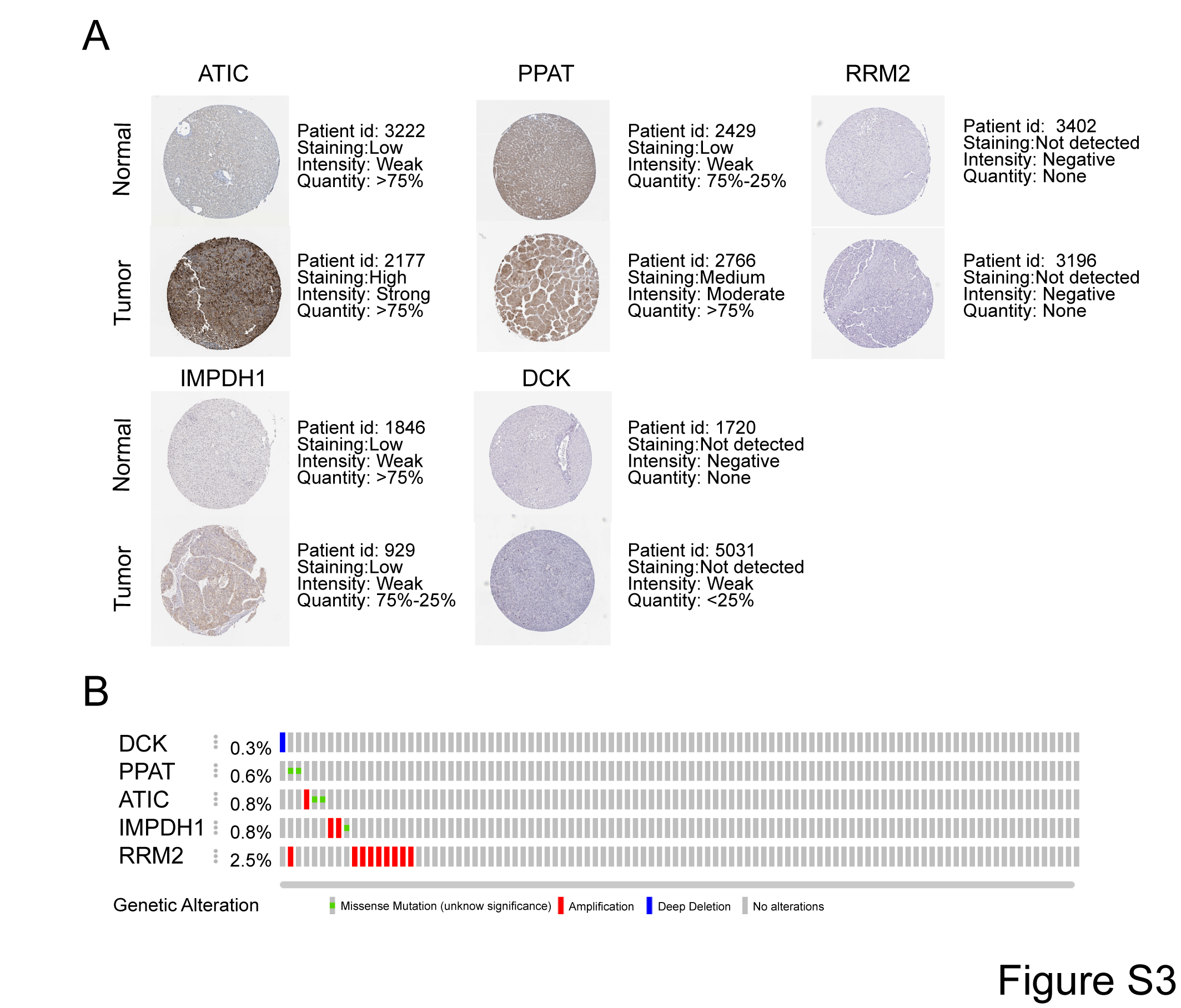

Supplement: Supplementary file 4 [file Image_3.tif]

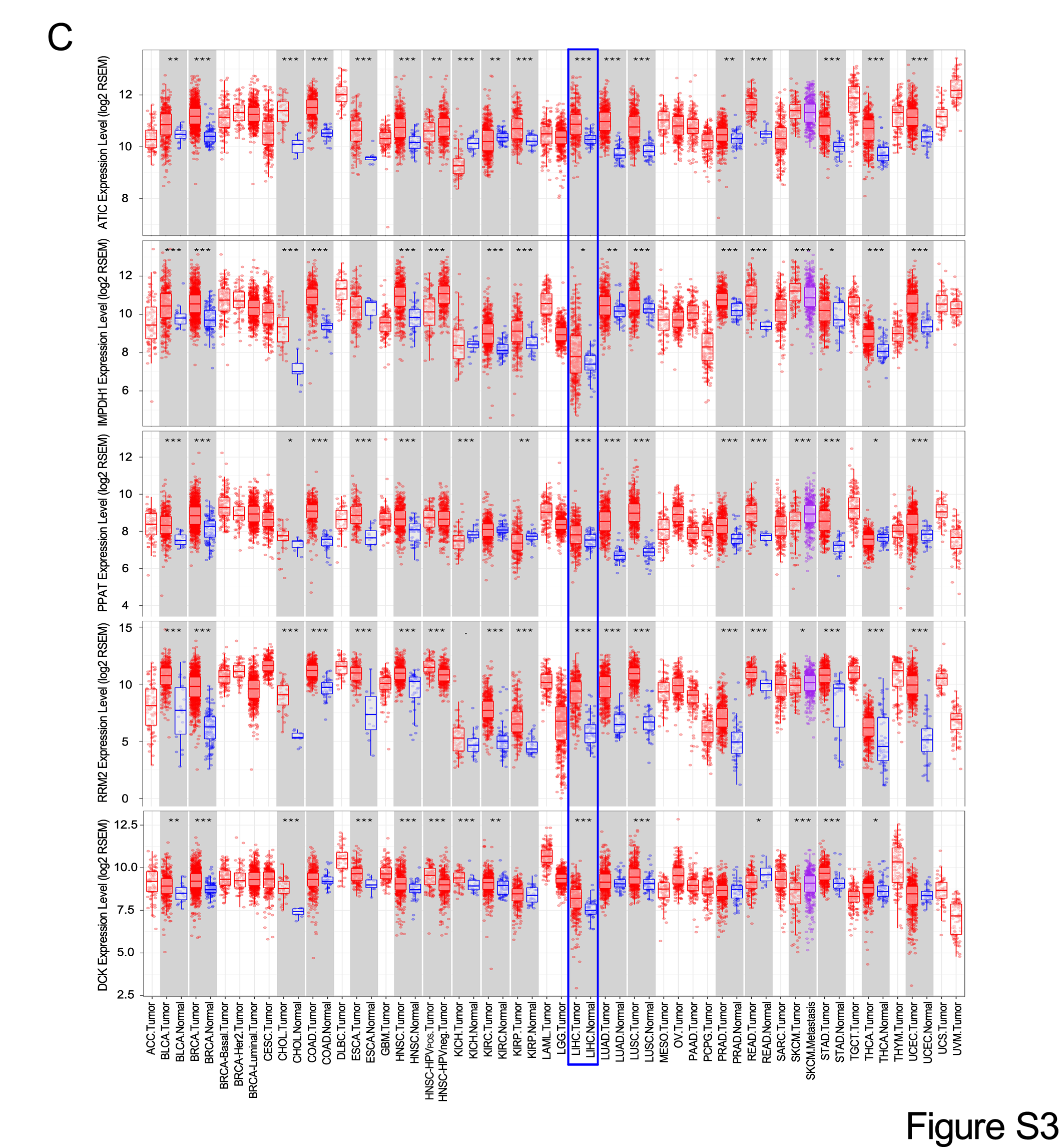

Supplement: Supplementary file 5 [file Image_4.tif]
